# Retraction Note: Nano-selenium supplementation: improving growth, digestibility and mineral absorption in freshwater fish, *Catla catla*

**DOI:** 10.1186/s12917-026-05830-z

**Published:** 2026-08-15

**Authors:** Nisar Ahmad, Syed Makhdoom Hussain, Shafaqat Ali, Muhammad Farrukh Tahir, Pallab K. Sarker, Mudassar Shahid

**Affiliations:** 1 Department of Zoology, University of Jhang, Syed Makhdoom Hussain, Jhang, 35200 Pakistan; 2 Fish Nutrition Laboratory, Department of Zoology, Government College University, Faisalabad, Faisalabad, 38000 Pakistan; 3 Department of Environmental Sciences, Government College University Faisalabad, Faisalabad, 38000 Pakistan; 4 Department of Biological Sciences and Technology, China Medical University, Taichung, 40402 Taiwan; 5 Department of Biochemistry, University of Jhang, Jhang, 35200 Pakistan; 6 Environmental Studies Department, University of California Santa Cruz, Santa Cruz, CA 95060 USA; 7 Department of Pharmaceutics, College of Pharmacy, King Saud University, Riyadh, 11451 Saudi Arabia

**Retraction Note: BMC Vet Rese 20, 438 (2024)**

**https://doi.org/10.1186/s12917-024-04291-6**

The Editor has retracted this article because there are significant scientific concerns and lack of clarity in key aspects of the methodology and data presentation, as well as language inconsistencies. There are also concerns of substantial overlaps with previously published articles [1, 2]. Additionally, the authorship of this paper could not be verified. Pallab K. Sarker has stated that he had played no role in the creation of this article. As a result, the scientific integrity of the article cannot be guaranteed.

Syed Makhdoom Hussain has stated on behalf of all the co-authors that they do not agree to this retraction.
